# Heavy Metal Pollution in a Soil-Rice System in the Yangtze River Region of China

**DOI:** 10.3390/ijerph13010063

**Published:** 2015-12-22

**Authors:** Zhouping Liu, Qiaofen Zhang, Tiqian Han, Yanfei Ding, Junwei Sun, Feijuan Wang, Cheng Zhu

**Affiliations:** College of Life Sciences, China Jiliang University, Hangzhou 310018, China; jslzp89@163.com (Z.L.); zhangqf025@163.com (Q.Z.); hantq96@163.com (T.H.); dingyanfei1984@126.com (Y.D.); Juville@cjlu.edu.cn (J.S.)

**Keywords:** soil-rice system, pollution characteristics, heavy metal accumulation, Yangtze River region

## Abstract

Heavy metals are regarded as toxic trace elements in the environment. Heavy metal pollution in soil or rice grains is of increasing concern. In this study, 101 pairs of soil and rice samples were collected from the major rice-producing areas along the Yangtze River in China. The soil properties and heavy metal (*i.e.*, Cd, Hg, Pb and Cr) concentrations in the soil and rice grains were analyzed to evaluate the heavy metal accumulation characteristics of the soil-rice systems. The results showed that the Cd, Hg, Pb and Cr concentrations in the soil ranged from 0.10 to 4.64, 0.01 to 1.46, 7.64 to 127.56, and 13.52 to 231.02 mg·kg^−^^1^, respectively. Approximately 37%, 16%, 60% and 70% of the rice grain samples were polluted by Cd, Hg, Pb, and Cr, respectively. The degree of heavy metal contamination in the soil-rice systems exhibited a regional variation. The interactions among the heavy metal elements may also influence the migration and accumulation of heavy metals in soil or paddy rice. The accumulation of heavy metals in soil and rice grains is related to a certain extent to the pH and soil organic matter (SOM). This study provides useful information regarding heavy metal accumulation in soil to support the safe production of rice in China. The findings from this study also provide a robust scientific basis for risk assessments regarding ecological protection and food safety.

## 1. Introduction

Heavy metals are regarded as nonessential and toxic trace elements in the environment [1]. Due to their potential toxicity, as well as their persistent and irreversible accumulation characteristics, heavy metals, such as Cd, Pb, Hg, and Cr, are listed as key monitoring pollutants by the Chinese Ministry of Environmental Protection [2]. Cr is classified as a priority pollutant in Category A (human carcinogen), while Cd and Pb are classified in Category B (probable human carcinogen) by the United States Environmental Protection Agency [3]. Soil is the most important reservoir of heavy metals in the terrestrial ecosystem, and the content of heavy metals in soils is an important indicator of environmental quality [4,5]. Soils are also the main source of heavy metals in plants, where they become the main source of heavy metals in plant-derived foods [6]. Heavy metals that accumulate and migrate in soil not only influence plant growth and yield, but also have the potential to accumulate in the human body through the food chain, thus posing a serious threat to human health [7,8,9]. Recently, the issue of heavy metal pollution in agricultural soils and plants has become increasingly serious [10,11,12]. Paddy rice is one of the main global food sources, and China is the largest producer of rice in the world; thus, the contamination and accumulation of heavy metals, such as Cd, Pb, Hg, and Cr, in rice grains have become a topic of great public concern [12,13]. Numerous studies have been performed on Cd, Pb, Cr, and Hg in soils or rice [13,14,15]. In China, approximately 10% of planting land is contaminated by heavy metals (Cd, Pb, Hg, Cr, *etc.*), and approximately ten million tons of grains are contaminated every year [14]. In a random survey, researchers discovered that 10% of the commercially available rice grain in China had excessive levels of Cd [15].

As one of the fastest developing regions in China, the Yangtze River region is also famous for being the “Home of Rice and Fish”. Due to its vast and fertile farmlands, the Yangtze River region, including Hubei, Hunan and Jiangxi Provinces, is China’s main rice-growing area. In recent years, the heavy metal pollution in rice grains and rice products has become increasingly serious in some parts of the Yangtze River region [12,13,16,17]. Cd, Pb, and Cr contamination in paddy soils from Hubei Province [16] and Cd-polluted rice in Hunan Province [17] have been reported. 

Researchers have performed numerous surveys and experiments regarding the sources of heavy metal pollution, the bioavailability of metals, and other issues in agricultural soil or plants [16,17,18,19]. However, most previous studies on the accumulation of heavy metals in rice were performed using pot or field experiments or focused on special use areas (e.g., mining areas). Due to the complexity of soil-plant relationships and the heterogeneity of a natural agricultural system, regional field investigations should be encouraged due to their greater practical significance. Moreover, there is a lack of information related to the migration and accumulation characteristics of heavy metal trace elements in soil-rice systems of a natural agricultural system and a lack of information about the factors (soil pH and soil organic matter) that directly influence the migration and accumulation of heavy metals in natural soil-rice systems. 

Therefore, a regional-scale survey was performed to study Cd, Pb, Hg, and Cr contamination and their influence factors in a soil-rice system under actual field conditions. The aims of this study were to: (1) study the accumulation status of heavy metals (Cd, Pb, Cr, and Hg) in soils and the corresponding rice grain and establish a corresponding traceable database; (2) study the relationships between the heavy metals (Cd, Pb, Cr, and Hg) in rice grain and the corresponding soils; and (3) clarify the factors (soil pH and soil organic matter) that control heavy metal accumulation in soil and rice grain.

## 2. Experimental Section

### 2.1. Sample Collection and Study Area

**Figure 1:**
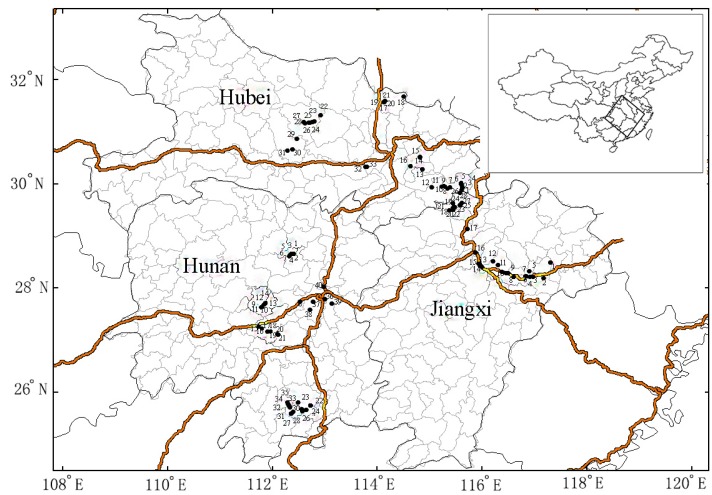
Distribution of sampling sites in the Yangtze River region (Hubei, Hunan and Jiangxi). The sampling areas in Hubei.: 1–6 Wuxue City, 7–12 Yangxin City, 13–16 Ezhou City, 121 Dawu City, 228 Zhongxiang City, 29–31 Shayang City, 32–33 Wuhan City; the sampling areas in Hunan: 1–7 Yiyang City, 8–14 Liangyu City, 15–18 Shaodong City, 121 Yiyang City, 22–26 Guiyang City, 27–30 Jiahe City, 31–35 Gongzhou City, 36 Zhuzhou City, 37–38 Xiangtan City, 39–40 Changsha City; the sampling areas in Jiangxi: 1–2 Guixi City, 3–6 Yujiang City, 7–8 Dongxiang City, 9–12 Jinxian City, 13–16 Nanchang City, 17–18 Yongxiu City, 19–28 Ruichang City.

Pairs of agricultural soil and rice grain samples (101) of were collected from large, thriving, representative paddy fields at the mature stage of rice growth along the midstream and downstream reaches of the Yangtze River in Hubei, Hunan, and Jiangxi Provinces (Figure 1). All the samples were collected in October 2012.

We designed a suitable route, chose the sampling areas, and went to the rice planting areas that were generally located between two towns. We collected three to six samples of soil and rice grain at each sampling point. Five to ten mature rice panicles were harvested from each paddy field. Soil samples (approximately 300 g) were collected from 10 to 15 cm above the surface from the same paddy fields.

### 2.2. Quality of the Applied Methodology

Soil samples were air-dried indoors at room temperature. They were crushed with a wooden stick, then passed through a 2.5 mm nylon sieve to remove grit and plant residues and then were finely ground with an agate mortar. They were screened through a 100-mesh nylon sieve, and a subsample (100 g) was bagged and stored in a dry location for heavy metal concentration and soil property analyses. Simultaneously, the samples of rice grain were rinsed with tap water, washed with distilled water and dried at 105 °C for 1 h and then at 70 °C to a constant weight in the oven. They were then shelled with a rice husking machine and ground to a powder for further analysis.

Soil samples were digested in a HNO_3_/HF mixture (3:1) in a microwave digester (CEM-MARS: Boston, MA, USA). Approximately 0.2 g of each of the mentioned soil samples was weighed and placed into a digestion vessel. High purity HNO_3_ (6 mL) and high purity HF (2 mL) were added to the vessels, which were then placed in a ventilation cabinet for 2–3 h. The samples were then digested in the microwave digester. Simultaneously, rice grain samples were digested using HNO_3_ and H_2_O_2_. Rice grain samples (0.3 g) were weighed and placed into digestion vessels. The vessels were stored in a ventilation cabinet for 2–3 h after 6 mL high purity HNO_3_ and 0.2 mL H_2_O_2_ were added, and then digested in the microwave digester. After digestion, all the mixtures were reduced to 1 mL at high temperature. The solutions were then diluted into 25 mL volumetric flasks with ultra-pure water, and the clarified samples thus obtained were stored in the refrigerator at 4 °C for further analysis.

The concentrations of the four heavy metals in soil and rice grain were measured using two different methods. Cd, Pb, and Cr concentrations were measured by graphite furnace atomic absorption spectrometry (AA7000, SHIMADZU: Kyoto, Japan); Hg concentrations were determined with hydride generation atomic absorption spectrometry (AA7000, SHIMADZU: Kyoto, Japan). All samples were measured in triplicate. Reagent blanks, a standard reference soil sample (GBW07443) and standard plant samples (GBW10010), obtained from the Centre of Standard Materials of China, were used to monitor the determination quality of the soil and rice samples, respectively. The recovery of samples spiked with standards ranged from 89% to 101.4%. The detection limits for Cd, Pb, Cr, and Hg were 0.0025, 0.027, 0.005, and 0.0002 µg·L^−1^, respectively. Soil pH was measured using the glass electrode method of Chaturvedi and Sankar [20], with a water to soil ratio of 2.5:1. Soil organic matter (SOM) content was determined by the chromic acid titration method [21].

### 2.3. Evaluation Standards for Soil and Rice Pollution

The Environmental Quality Standards for Soils in China [22] and heavy metal standards for food safety [23] (Table 1) were used as guideline values for the assessment of heavy metal accumulation and pollution in soil and rice grains. The Environmental Quality Standards for Soils includes three levels, and the second one was adopted as the guideline value for agricultural production.

**Table 1:** Environmental quality standard for soils in China (GB 15618-1995) [22], heavy metal standards for food safety (GB2715–2005) [23] (mg·kg^−1^).

Heavy Metals	Grade 1	Grade 2			Grade 3	Food Safety Standards (In Rice)
	National Backgrounds	pH < 6.5	pH 6.5–pH 7.5	pH > 7.5	pH > 6.5	
Cd ≤	0.20	0.30	0.30	0.6	1.0	0.20
Hg ≤	0.15	0.30	0.50	1.0	1.5	0.02
Pb ≤	35	250	300	350	500	0.20
Cr ≤	90	250	300	350	400	1.0

### 2.4. Statistical Analysis

A standard statistical analysis (mean, standard deviation, *etc.*) was performed to describe soil properties (SOM and pH) in three study areas. Significant differences among areas required evaluation by ANOVA and Kruskal–Wallis tests (α  =  0.01). To study the relationship between soil element pollution and rice element contents, canonical correlation analyses (CCorA) were used. The location and sample map was produced using the ArcGIS 10.0 software (ESRI: Redlands, CA, USA). All statistical analysis data were statistically analyzed by the XLSTAT (Addinsoft: Brooklyn, NY, USA) and SPSS 18.0 programs (IBM: Chicago, IL, USA).

## 3. Results and Discussion

### 3.1. Heavy Metal Contamination in Soil and Soil Properties

The concentrations of heavy metals in soils from Hubei, Hunan and Jiangxi Provinces are presented in Table 2. Compared with the Environmental Quality Standards for Soils in China [22] (Table 1), 78.78%, 6.06%, 3.03% and 21.21% of the 33 soil samples from Hubei Province exceeded the local soil national backgrounds for Cd, Hg, Pb and Cr, respectively, which indicated that some heavy metals had accumulated in the surveyed soil. Combined with the corresponding pH values shown in Table 2, among the 33 sampling points from Hubei Province, 45.45% and 75.76% of the soil samples exceeded the Grade 2 thresholds for Cd and Hg, respectively, whereas the Pb and Cr concentrations of soil samples from Hubei Province were under the Grade 2 thresholds.

In Hunan Province, 92.5%, 27.5%, 15% and 10% of the 40 surveyed soil samples exceeded the local soil backgrounds for Cd, Hg, Pb and Cr, respectively, indicating that Cd had accumulated considerably in the surveyed soil. Combined with the corresponding pH values, except for the samples at sites 6, 16 and 17, the remaining soil samples exceeded the Grade 2 thresholds for Cd, and some samples exceeded the Grade 3 thresholds. Only 7.5% of the tested soil samples were not contaminated by Cd. Three soil samples (7.5%) exceeded the Grade 2 thresholds for Hg. The Pb and Cr concentrations in soil samples from Hunan Province were under the Grade 2 thresholds.

Similarly, in the 28 soil samples from Jiangxi Province, 78.57%, 25%, 7.14% and 32.14% of the samples had Cd, Hg, Pb and Cr concentrations exceeding the local soil backgrounds, respectively. As shown in Table 2, the Cd concentration of 19 soil samples (67.8%) exceeded the Grade 2 thresholds. Two soil samples showed Hg concentrations exceeding the Grade 2 thresholds, whereas all samples had Pb and Cr concentrations below the Grade 2 thresholds.

**Table 2:** Soil properties and heavy metal concentrations (Cd, Hg, Pb, Cr) in the soil and rice of Hubei, Hunan and Jiangxi.

	Location	Soil						Rice Grain			
		Cd (mg·kg^−1^)	Hg (mg·kg^−1^)	Pb (mg·kg^−1^)	Cr (mg·kg^−1^)	pH	SOM (mg·kg^−1^)	Cd (mg·kg^−1^)	Hg (mg·kg^−1^)	Pb (mg·kg^−1^)	Cr (mg·kg^−1^)
**Hubei**	1	0.19 ± 0.05	1.46 ± 0.13	27.95 ± 1.11	212.19 ± 20.68	5.83 ± 0.05	19.26 ± 1.38	0.03 ± 0.02	0.015 ± 0.003	0.18 ± 0.05	1.09 ± 0.22
	2	0.66 ± 0.09	0.68 ± 0.12	16.8 ± 1.12	42.87 ± 2.56	6.41 ± 0.03	27.52 ± 1.06	0.05 ± 0.03	0.017 ± 0.002	0.18 ± 0.06	0.65 ± 0.10
	3	0.21 ± 0.04	0.49 ± 0.06	18.27 ± 1.34	49.49 ± 9.08	6.09 ± 0.25	20.64 ± 2.10	0.02 ± 0.00	0.018 ± 0.002	0.23 ± 0.06	0.64 ± 0.16
	4	0.31 ± 0.01	0.37 ± 0.05	18.27 ± 1.56	47.82 ± 10.22	4.46 ± 0.44	20.64 ± 1.78	0.17 ± 0.03	0.002 ± 0.001	0.15 ± 0.03	0.62 ± 0.20
	5	0.35 ± 0.04	0.49 ± 0.04	32.61 ± 2.46	53.79 ± 8.87	6.14 ± 0.23	13.76 ± 0.67	0.06 ± 0.02	0.001 ± 0.000	0.30 ± 0.09	0.8 ± 0.15
	6	0.31 ± 0.03	0.6 ± 0.08	20.15 ± 1.23	34.43 ± 3.22	4.75 ± 0.55	27.52 ± 1.99	0.14 ± 0.05	0.001 ± 0.000	0.21 ± 0.06	0.68 ± 0.11
	7	0.95 ± 0.10	0.77 ± 0.10	29.37 ± 2,78	224.99 ± 45.38	6.13 ± 0.04	27.52 ± 0.34	0.09 ± 0.02	0.001 ± 0.000	0.20 ± 0.04	33.82 ± 6.29
	8	0.43 ± 0.09	0.49 ± 0.08	27.91 ± 1.34	114.54 ± 26.11	5.98 ± 0.02	28.89 ± 0.98	0.04 ± 0.01	0.000 ± 0.000	0.26 ± 0.03	0.52 ± 0.12
	9	0.51 ± 0.04	0.72 ± 0.29	36.48 ± 3.12	231.02 ± 11.98	6.86 ± 0.06	41.96 ± 7.10	0.04 ± 0.02	0.002 ± 0.000	0.20 ± 0.03	29.89 ± 6.98
	10	0.82 ± 0.11	0.69 ± 0.18	34.35 ± 2.45	220.04 ± 34.39	6.62 ± 0.12	39.21 ± 6.02	0.03 ± 0.00	0.007 ± 0.001	0.19 ± 0.08	1.32 ± 0.18
	11	0.88 ± 0.12	0.7 ± 0.21	24.52 ± 2.45	38.73 ± 4.56	6.8 ± 0.22	41.27 ± 9.12	0.04 ± 0.01	0.01 ± 0.003	0.19 ± 0.07	0.87 ± 0.15
	12	0.41 ± 0.10	0.51 ± 0.18	32.88 ± 5.45	38.49 ± 1.89	5.98 ± 0.68	20.64 ± 2.89	0.13 ± 0.06	0.011 ± 0.002	0.21 ± 0.10	0.81 ± 0.07
	13	0.82 ± 0.11	0.90 ± 0.34	34.38 ± 6.86	24.05 ± 2.43	6.76 ± 0.24	30.95 ± 3.56	0.03 ± 0.00	0.011 ± 0.002	0.30 ± 0.01	0.59 ± 0.15
	14	0.44 ± 0.07	0.44 ± 0.11	20.76 ± 1.28	45.2 ± 6.56	6.91 ± 0.41	16.51 ± 1.64	0.04 ± 0.01	0.011 ± 0.002	0.19 ± 0.05	7.01 ± 3.22
	15	0.17 ± 0.01	0.21 ± 0.09	15.47 ± 1.50	47.15 ± 2.44	7.02 ± 0.06	12.31 ± 2.37	0.02 ± 0.00	0.012 ± 0.002	0.35 ± 0.08	1.10 ± 0.37
	16	0.3 ± 0.10	0.37 ± 0.05	32.73 ± 3.88	41.76 ± 3.51	6.7 ± 0.34	18.57 ± 1.88	0.04 ± 0.02	0.015 ± 0.004	0.28 ± 0.03	1.22 ± 0.34
	17	0.25 ± 0.09	0.22 ± 0.01	14.54 ± 3.11	34.93 ± 1.45	4.94 ± 0.08	14.45 ± 1.28	0.20 ± 0.09	0.011 ± 0.001	0.27 ± 0.09	0.90 ± 0.16
	18	0.41 ± 0.13	0.46 ± 0.17	16.82 ± 4.80	35.77 ± 2.22	6.09 ± 0.27	19.95 ± 3.56	0.10 ± 0.04	0.016 ± 0.003	0.19 ± 0.05	0.74 ± 0.21
	19	0.36 ± 0.08	0.35 ± 0.16	15.61 ± 4.41	42.95 ± 5.10	6.00 ± 0.06	12.38 ± 0.61	0.08 ± 0.02	0.014 ± 0.003	0.13 ± 0.01	5.91 ± 1.11
	20	0.49 ± 0.07	0.39 ± 0.10	13.18 ± 1.28	39.84 ± 1.00	6.21 ± 0.06	41.27 ± 4.14	0.16 ± 0.01	0.022 ± 0.01	0.13 ± 0.02	9.84 ± 0.60
	21	1.24 ± 0.10	0.29 ± 0.01	21.03 ± 2.12	213.29 ± 11.38	6.19 ± 0.04	32.33 ± 2.09	0.02 ± 0.00	0.027 ± 0.004	0.23 ± 0.03	38.96 ± 0.98
	22	0.22 ± 0.07	0.43 ± 0.08	21.55 ± 0.98	64.11 ± 4.50	6.06 ± 0.05	21.32 ± 1.32	0.10 ± 0.00	0.009 ± 0.002	0.19 ± 0.02	4.07 ± 0.22
	23	0.27 ± 0.09	0.63 ± 0.09	17.78 ± 0.26	48.17 ± 4.92	6.43 ± 0.31	16.51 ± 0.98	0.04 ± 0.01	0.001 ± 0.001	0.22 ± 0.02	2.42 ± 0.52
	24	0.29 ± 0.07	0.46 ± 0.08	24.02 ± 0.67	46.58 ± 3.30	6.26 ± 0.38	27.52 ± 1.58	0.06 ± 0.01	0.009 ± 0.001	0.18 ± 0.01	1.89 ± 0.34
	25	0.18 ± 0.10	0.38 ± 0.06	30.02 ± 2.33	62.83 ± 4.42	6.30 ± 0.28	12.38 ± 1.04	0.05 ± 0.00	0.019 ± 0.006	0.27 ± 0.02	6.60 ± 0.51
	26	0.10 ± 0.01	0.57 ± 0.11	21.3 ± 1.04	83.18 ± 3.67	6.20 ± 0.40	7.57 ± 0.88	0.09 ± 0.01	0.016 ± 0.004	0.25 ± 0.01	9.90 ± 1.02
	27	0.20 ± 0.01	0.33 ± 0.08	23.41 ± 1.48	46.17 ± 3.08	6.41 ± 0.08	22.01 ± 3.13	0.04 ± 0.01	0.015 ± 0.00	0.23 ± 0.01	6.01 ± 0.80
	28	0.26 ± 0.01	0.5 ± 0.03	17.17 ± 0.34	43.18 ± 5.10	6.31 ± 0.08	15.82 ± 1.34	0.07 ± 0.02	0.018 ± 0.007	0.29 ± 0.03	2.15 ± 0.06
	29	0.76 ± 0.04	0.32 ± 0.03	17.82 ± 0.67	42.4 ± 2.88	5.37 ± 0.06	19.26 ± 1.02	0.15 ± 0.01	0.017 ± 0.003	0.36 ± 0.04	3.06 ± 0.50
	30	0.32 ± 0.02	0.42 ± 0.07	18.42 ± 1.45	220.63 ± 13.09	4.24 ± 0.04	20.64 ± 1.34	0.40 ± 0.08	0.027 ± 0.004	0.19 ± 0.01	2.85 ± 0.08
	31	0.25 ± 0.01	0.32 ± 0.10	17.28 ± 0.72	40.89 ± 1.06	4.13 ± 0.07	15.13 ± 0.90	0.49 ± 0.03	0.006 ± 0.002	0.24 ± 0.01	1.92 ± 0.22
	32	0.47 ± 0.09	0.22 ± 0.05	23.96 ± 1.06	68.93 ± 4.26	6.69 ± 0.42	21.32 ± 1.38	0.08 ± 0.04	0.011 ± 0.002	0.17 ± 0.01	1.80 ± 0.19
	33	0.49 ± 0.06	0.19 ± 0.02	27.78 ± 0.45	80.48 ± 5.72	6.64 ± 0.02	33.02 ± 2.64	0.08 ± 0.01	0.01 ± 0.005	0.19 ± 0.01	1.45 ± 0.06
**Hunan**	1	0.55 ± 0.10	0.01 ± 0.01	25.08 ± 2.34	140.7 ± 8.38	4.11 ± 0.12	30.27 ± 2.81	0.68 ± 0.12	0.007 ± 0.001	0.21 ± 0.03	0.53 ± 0.10
	2	3.44 ± 0.22	0.03 ± 0.00	22.2 ± 1.02	53.74 ± 3.46	6.82 ± 0.06	49.53 ± 3.54	0.25 ± 0.01	0.011 ± 0.01	0.20 ± 0.02	0.77 ± 0.08
	3	0.50 ± 0.04	0.05 ± 0.00	20.08 ± 0.49	61.82 ± 2.85	3.90 ± 0.10	23.39 ± 4.05	1.36 ± 0.13	0.013 ± 0.005	0.21 ± 0.01	0.41 ± 0.09
	4	0.47 ± 0.04	0.18 ± 0.02	19.21 ± 1.21	65.71 ± 5.24	5.91 ± 0.20	30.27 ± 3.18	0.10 ± 0.01	0.011 ± 0.004	0.21 ± 0.01	0.49 ± 0.01
	5	0.79 ± 0.03	0.06 ± 0.02	22.89 ± 3.44	64.62 ± 6.06	4.85 ± 0.15	46.78 ± 6.02	0.44 ± 0.01	0.019 ± 0.005	0.18 ± 0.02	0.35 ± 0.05
	6	0.29 ± 0.00	0.05 ± 0.01	25.65 ± 2.05	40.75 ± 3.56	6.51 ± 0.07	12.38 ± 3.11	0.24 ± 0.03	0.018 ± 0.005	0.17 ± 0.01	6.32 ± 0.90
	7	1.64 ± 0.10	0.03 ± 0.01	19.99 ± 1.05	68.54 ± 2.69	6.08 ± 0.12	39.9 ± 1.89	0.03 ± 0.01	0.019 ± 0.01	0.29 ± 0.01	0.47 ± 0.05
	8	0.97 ± 0.06	0.26 ± 0.05	21.56 ± 2.18	61.15 ± 3.06	6.30 ± 0.08	33.02 ± 2.34	1.02 ± 0.06	0.015 ± 0.002	0.49 ± 0.08	7.64 ± 0.03
	9	0.59 ± 0.04	0.07 ± 0.03	29.81 ± 0.88	82.65 ± 3.12	5.00 ± 0.06	30.27 ± 4.20	0.02 ± 0.00	0.017 ± 0.001	0.39 ± 0.04	3.30 ± 0.08
	10	1.46 ± 0.14	0.31 ± 0.06	15.15 ± 0.45	89.35 ± 5.15	6.95 ± 0.07	57.78 ± 7.44	0.09 ± 0.01	0.007 ± 0.001	0.36 ± 0.02	3.21 ± 0.20
	11	1.94 ± 0.09	0.20 ± 0.03	38.26 ± 2.65	33.43 ± 2.83	6.40 ± 0.03	45.4 ± 2.08	0.03 ± 0.00	0.011 ± 0.006	0.68 ± 0.06	3.67 ± 0.11
	12	3.44 ± 0.67	0.10 ± 0.02	23.67 ± 1.67	43.25 ± 2.04	6.19 ± 0.44	50.9 ± 2.42	0.115 ± 0.01	0.005 ± 0.001	0.36 ± 0.05	3.49 ± 0.09
	13	0.32 ± 0.04	0.04 ± 0.01	23.39 ± 3.81	83.41 ± 6.72	6.13 ± 0.50	19.26 ± 1.09	0.065 ± 0.01	0.006 ± 0.001	0.28 ± 0.02	3.42 ± 0.31
	14	0.70 ± 0.11	0.14 ± 0.03	11.44 ± 0.56	64.56 ± 4.38	6.86 ± 0.20	22.01 ± 0.84	0.02 ± 0.00	0.007 ± 0.002	0.48 ± 0.02	2.84 ± 0.42
	15	1.08 ± 0.42	0.19 ± 0.02	25.56 ± 1.89	75.97 ± 5.47	6.61 ± 0.04	38.52 ± 2.55	0.03 ± 0.01	0.006 ± 0.002	0.38 ± 0.02	2.97 ± 0.36
	16	0.17 ± 0.02	0.11 ± 0.01	23.53 ± 0.85	41.53 ± 2.70	6.7 ± 0.08	8.25 ± 1.00	0.01 ± 0.00	0.009 ± 0.003	0.44 ± 0.04	2.67 ± 0.04
	17	0.30 ± 0.05	0.04 ± 0.01	19.61 ± 2.31	73.7 ± 4.36	6.73 ± 0.06	8.25 ± 0.48	0.07 ± 0.01	0.013 ± 0.005	0.21 ± 0.01	2.51 ± 0.06
	18	0.71 ± 0.0	0.01 ± 0.00	32.39 ± 3.58	77.21 ± 2.99	6.28 ± 0.12	39.90 ± 2.56	0.03 ± 0.00	0.015 ± 0.005	0.30 ± 0.04	2.56 ± 0.05
	19	0.45 ± 0.10	0.06 ± 0.01	24.15 ± 4.08	56.11 ± 4.00	6.20 ± 0.44	24.76 ± 1.08	0.19 ± 0.05	0.013 ± 0.006	0.33 ± 0.08	2.54 ± 0.07
	20	1.46 ± 0.62	0.1 ± 0.05	26.89 ± 1.67	78.4 ± 3.98	6.11 ± 0.54	30.27 ± 2.22	0.62 ± 0.14	0.008 ± 0.003	0.39 ± 0.03	2.82 ± 0.16
	21	2.38 ± 0.56	0.03 ± 0.01	32.72 ± 3.28	69.02 ± 17.45	5.39 ± 0.48	17.88 ± 0.67	0.77 ± 0.21	0.019 ± 0.006	0.56 ± 0.05	3.41 ± 0.67
	22	3.34 ± 0.33	0.11 ± 0.05	127.56 ± 6.82	62.14 ± 6.12	6.14 ± 0.14	39.9 ± 2.28	0.18 ± 0.01	0.016 ± 0.005	0.38 ± 0.03	4.72 ± 1.16
	23	1.27 ± 0.25	0.13 ± 0.0	21.28 ± 1.62	70.41 ± 3.88	6.10 ± 0.12	45.4 ± 5.67	1.46 ± 0.03	0.014 ± 0.007	0.43 ± 0.02	4.22 ± 0.86
	24	0.57 ± 0.04	0.79 ± 0.0	89.37 ± 4.50	59.41 ± 4.21	5.86 ± 0.08	26.14 ± 1.32	0.73 ± 0.04	0.006 ± 0.001	0.44 ± 0.04	4.59 ± 0.42
	25	1.14 ± 0.08	0.01 ± 0.00	21.82 ± 2.00	32.86 ± 5.38	5.77 ± 0.14	44.02 ± 3.52	0.014 ± 0.01	0.021 ± 0.007	0.27 ± 0.01	4.80 ± 0.61
	26	1.06 ± 0.06	0.11 ± 0.03	13.39 ± 0.56	60.45 ± 3.45	6.11 ± 0.16	38.52 ± 2.65	0.03 ± 0.01	0.023 ± 0.008	0.31 ± 0.05	4.32 ± 0.35
	27	0.38 ± 0.02	0.01 ± 0.00	10.47 ± 0.99	77.19 ± 6.11	5.93 ± 0.48	24.76 ± 1.28	0.04 ± 0.01	0.021 ± 0.001	0.23 ± 0.01	0.33 ± 0.01
	28	0.47 ± 0.08	0.07 ± 0.01	21.21 ± 3.45	49.93 ± 5.45	4.83 ± 0.16	41.27 ± 4.45	0.05 ± 0.01	0.013 ± 0.001	0.31 ± 0.01	4.47 ± 0.60
	29	0.37 ± 0.09	0.29 ± 0.07	30.02 ± 6.98	37.37 ± 3.22	5.91 ± 0.18	17.88 ± 1.65	0.05 ± 0.00	0.022 ± 0.002	0.18 ± 0.01	0.23 ± 0.02
	30	0.35 ± 0.08	0.08 ± 0.02	12.8 ± 1.78	67.58 ± 5.45	6.18 ± 0.40	34.39 ± 3.55	0.044 ± 0.01	0.024 ± 0.003	0.29 ± 0.00	4.20 ± 0.29
	31	0.49 ± 0.16	0.14 ± 0.0	16.29 ± 0.99	80.35 ± 6.68	6.34 ± 0.12	30.27 ± 2.86	0.02 ± 0.01	0.024 ± 0.003	0.40 ± 0.08	4.09 ± 0.82
	32	1.12 ± 0.09	0.17 ± 0.00	19.17 ± 1.34	39.17 ± 1.67	6.19 ± 0.18	26.14 ± 1.64	0.02 ± 0.00	0.009 ± 0.002	0.23 ± 0.02	0.64 ± 0.08
	33	0.62 ± 0.14	0.63 ± 0.08	36.57 ± 7.22	78.45 ± 9.02	5.88 ± 0.20	16.51 ± 1.12	0.09 ± 0.01	0.009 ± 0.002	0.31 ± 0.02	3.95 ± 1.23
	34	1.38 ± 0.32	0.15 ± 0.02	52.69 ± 6.12	84.26 ± 6.12	6.25 ± 0.14	44.02 ± 4.34	0.42 ± 0.08	0.01 ± 0.002	0.42 ± 0.04	71.04 ± 9.08
	35	2.83 ± 0.23	0.06 ± 0.00	29.49 ± 4.56	77.46 ± 2.55	5.98 ± 0.08	48.15 ± 2.85	1.81 ± 0.31	0.028 ± 0.002	0.22 ± 0.01	5.92 ± 3.82
	36	0.64 ± 0.09	0.26 ± 0.04	59.8 ± 7.45	85.38 ± 4.33	5.18 ± 0.12	34.39 ± 2.66	1.03 ± 0.10	0.032 ± 0.003	0.32 ± 0.02	5.60 ± 1.08
	37	0.54 ± 0.12	0.3 ± 0.08	32.93 ± 2.34	75.52 ± 1.32	5.19 ± 0.08	26.14 ± 1.05	1.34 ± 0.09	0.017 ± 0.003	0.31 ± 0.05	4.25 ± 0.49
	38	0.71 ± 0.21	0.1 ± 0.03	17.18 ± 0.78	52.76 ± 4.34	5.91 ± 0.01	22.01 ± 1.87	1.64 ± 0.09	0.012 ± 0.01	0.49 ± 0.03	4.25 ± 1.02
	39	2.76 ± 0.30	0.6 ± 0.09	17.59 ± 1.56	140.45 ± 10.20	5.34 ± 0.22	34.39 ± 2.35	1.40 ± 0.06	0.014 ± 0.00	0.29 ± 0.02	4.12 ± 0.87
	40	1.19 ± 0.17	0.14 ± 0.05	27.21 ± 4.23	103.22 ± 6.72	5.66 ± 0.08	37.15 ± 2.44	0.03 ± 0.01	0.001 ± 0.000	0.19 ± 0.00	4.12 ± 0.65
**Jiangxi**	1	0.26 ± 0.04	0.19 ± 0.05	15.36 ± 1.22	47.05 ± 3.56	4.34 ± 0.04	13.76 ± 1.22	0.40 ± 0.08	0.005 ± 0.002	0.31 ± 0.02	2.34 ± 0.10
	2	1.22 ± 0.14	0.06 ± 0.02	13.47 ± 0.89	34.5 ± 10.28	4.36 ± 0.12	16.51 ± 1.56	0.15 ± 0.03	0.013 ± 0.002	0.23 ± 0.01	1.44 ± 0.08
	3	0.59 ± 0.08	0.22 ± 0.07	14.46 ± 0.67	62.86 ± 12.43	4.09 ± 0.06	7.57 ± 2.11	0.05 ± 0.01	0.015 ± 0.007	0.35 ± 0.02	1.05 ± 0.09
	4	0.60 ± 0.12	0.04 ± 0.01	7.64 ± 1.11	13.52 ± 5.02	4.16 ± 0.08	25.45 ± 3.65	0.21 ± 0.06	0.022 ± 0.004	0.25 ± 0.02	3.25 ± 0.11
	5	0.17 ± 0.04	0.09 ± 0.02	9.86 ± 2.34	100.88 ± 10.53	4.25 ± 0.12	15.13 ± 1.20	0.28 ± 0.02	0.021 ± 0.007	0.50 ± 0.08	59.93 ± 6.08
	6	0.36 ± 0.08	0.12 ± 0.01	12.00 ± 4.12	37.43 ± 1.67	3.79 ± 0.08	52.97 ± 7.02	0.10 ± 0.09	0.024 ± 0.004	0.36 ± 0.02	77.54 ± 11.01
	7	0.15 ± 0.04	0.16 ± 0.03	15.64 ± 3.34	138.54 ± 8.88	3.99 ± 0.20	9.63 ± 2.44	0.26 ± 0.04	0.011 ± 0.004	0.38 ± 0.03	6.45 ± 0.91
	8	1.24 ± 0.24	0.10 ± 0.02	11.14 ± 1.08	73.95 ± 4.94	4.09 ± 0.09	20.64 ± 1.06	0.19 ± 0.03	0.009 ± 0.002	0.25 ± 0.02	6.95 ± 1.08
	9	0.14 ± 0.04	0.15 ± 0.04	14.08 ± 2.78	114.07 ± 10.02	4.85 ± 0.11	28.2 ± 0.86	0.39 ± 0.06	0.014 ± 0.002	0.44 ± 0.04	3.16 ± 0.20
	10	0.14 ± 0.02	0.48 ± 0.13	18.54 ± 3.56	86.06 ± 3.98	4.84 ± 0.14	13.76 ± 0.29	0.89 ± 0.13	0.012 ± 0.004	0.28 ± 0.01	2.66 ± 0.48
	11	0.36 ± 0.10	0.17 ± 0.02	18.78 ± 3.17	110.06 ± 16.67	5.09 ± 0.22	35.77 ± 3.18	0.34 ± 0.08	0.006 ± 0.002	0.25 ± 0.03	1.58 ± 0.52
	12	0.15 ± 0.05	0.15 ± 0.01	16.91 ± 1.42	78.13 ± 4.24	4.90 ± 0.12	41.96 ± 1.67	0.09 ± 0.01	0.009 ± 0.001	0.61 ± 0.09	10.28 ± 2.00
	13	0.24 ± 0.06	0.14 ± 0.02	10.33 ± 1.29	93.52 ± 10.08	5.00 ± 0.04	26.83 ± 1.35	0.15 ± 0.03	0.03 ± 0.003	0.29 ± 0.02	93.74 ± 3.91
	14	0.24 ± 0.04	0.35 ± 0.07	19.69 ± 2.61	90.27 ± 4.67	6.2 ± 0.06	27.52 ± 0.79	0.10 ± 0.04	0.013 ± 0.003	0.22 ± 0.01	10.32 ± 1.18
	15	0.17 ± 0.07	0.15 ± 0.01	8.89 ± 1.52	43.53 ± 5.12	5.46 ± 0.09	29.58 ± 1.86	0.07 ± 0.03	0.008 ± 0.004	0.25 ± 0.02	2.23 ± 0.25
	16	0.71 ± 0.12	0.09 ± 0.02	12.81 ± 0.89	63.21 ± 12.44	5.54 ± 0.02	31.64 ± 3.22	0.06 ± 0.02	0.008 ± 0.001	0.32 ± 0.04	3.93 ± 0.72
	17	4.64 ± 0.51	0.06 ± 0.00	26.75 ± 1.28	57.53 ± 1.98	5.99 ± 0.04	34.39 ± 1.67	0.35 ± 0.06	0.004 ± 0.001	0.22 ± 0.02	1.23 ± 0.22
	18	2.66 ± 0.26	0.04 ± 0.00	25.74 ± 2.31	97.76 ± 7.51	5.78 ± 0.12	39.91 ± 6.08	0.22 ± 0.07	0.003 ± 0.000	0.12 ± 0.01	0.81 ± 0.13
	19	3.58 ± 1.28	0.13 ± 0.01	36.18 ± 4.72	90.95 ± 2.32	6.29 ± 0.17	38.52 ± 8.02	0.44 ± 0.09	0.09 ± 0.004	0.23 ± 0.01	0.78 ± 0.04
	20	2.40 ± 0.67	0.11 ± 0.00	27.72 ± 1.78	90.33 ± 7.15	5.84 ± 0.03	44.02 ± 4.25	0.64 ± 0.17	0.002 ± 0.001	0.15 ± 0.03	1.25 ± 0.32
	21	3.42 ± 0.22	0.06 ± 0.01	27.82 ± 3.45	86.6 ± 14.12	5.81 ± 0.15	52.28 ± 7.31	0.96 ± 0.21	0.005 ± 0.001	0.34 ± 0.02	1.35 ± 0.19
	22	1.43 ± 0.36	0.09 ± 0.00	28.49 ± 4.02	89.24 ± 10.58	5.88 ± 0.19	53.65 ± 7.04	0.67 ± 0.14	0.001 ± 0.000	0.17 ± 0.01	0.93 ± 0.06
	23	2.12 ± 0.48	0.04 ± 0.01	31.21 ± 3.33	68.05 ± 3.98	6.24 ± 0.03	35.77 ± 2.43	0.29 ± 0.05	0.003 ± 0.001	0.18 ± 0.04	1.05 ± 0.11
	24	0.64 ± 0.14	0.03 ± 0.00	21.7 ± 1.21	78.3 ± 5.82	5.51 ± 0.06	24.76 ± 2.89	0.42 ± 0.04	0.005 ± 0.001	0.22 ± 0.03	0.90 ± 0.06
	25	0.74 ± 0.12	0.06 ± 0.01	19.65 ± 0.99	78.32 ± 3.77	5.89 ± 0.14	46.78 ± 11.45	0.61 ± 0.06	0.004 ± 0.001	0.25 ± 0.05	0.76 ± 0.05
	26	1.42 ± 0.36	0.07 ± 0.01	24.12 ± 4.18	63.61 ± 9.22	6.04 ± 0.08	42.65 ± 4.88	0.37 ± 0.04	0.008 ± 0.003	0.23 ± 0.01	0.86 ± 0.06
	27	0.91 ± 0.21	0.05 ± 0.01	18.01 ± 3.09	55.37 ± 1.62	5.79 ± 1.02	30.27 ± 3.67	0.28 ± 0.07	0.001 ± 0.000	0.28 ± .002	0.77 ± 0.11
	28	2.86 ± 0.34	0.12 ± 0.02	54.64 ± 6.29	65.86 ± 6.08	5.39 ± 0.28	44.02 ± 5.23	0.52 ± 0.14	0.005 ± 0.001	0.28 ± 0.03	0.90 ± 0.09

Note: Results are represented as the mean ± standard deviation (*n* = 3 biological replicates); The sampling areas in **Hubei**: 1–6 Wuxue City, 7–12 Yangxin City, 13–16 Ezhou City, 17–21Dawu City, 22–28 Zhongxiang City, 29–31 Shayang City, 32–33 Wuhan City; the sampling areas in **Hunan**: 1–7 Yiyang City, 8–14 Liangyu City, 15–18 Shaodong City, 19–21 Yiyang City, 22–26 Guiyang City, 27–30 Jiahe City, 31–35 Gongzhou City 36 Zhuzhou City 37–38 Xiangtan City 39–40 Changsha City; the sampling areas in **Jiangxi**: 1–2 Guixi City, 3–6 Yujiang City, 7–8 Dongxiang City, 9–12 Jinxian City, 13–16 Nanchang City, 17–18 Yongxiu City, 19–28 Ruichang City.

The average concentrations of heavy metals in soil are presented in Table 3. The average concentrations of Cd, Hg, Pb, and Cr in soils from Hubei Province were 0.43, 0.50, 23.16, and 81.24 mg·kg^−1^, respectively. The average concentrations of Cd, Hg, Pb, and Cr in soils from Hunan Province were 1.12, 0.15, 29.00, and 69.00 mg·kg^−1^, respectively. The average concentrations of Cd, Hg, Pb, and Cr in soils from Jiangxi Province were 1.20, 0.13, 20.06, and 75.34 mg·kg^−1^, respectively. The Cd accumulation in soils among the test samples was in the order of Hunan = Jiangxi > Hubei; the Hg contamination of the soils was in order the of Hubei > Hunan = Jiangxi; the Pb concentration of the soils was in the order of Hunan > Hubei > Jiangxi; and the Cr contamination of the soils among the test samples from three provinces showed no significant difference. The results show that some soil samples from this region were polluted by Cd and Hg, and heavy metal contamination is serious at some sample sites. Cd and Hg contamination in agricultural soils may originate from: (1) atmospheric fallout from industrial and urban activities; (2) the application of sewage sludge and manure as fertilizer; and (3) the widespread use of organic and complex fertilizers [11,24,25,26]. Previous studies have shown that the application of K fertilizer significantly affects the Cd concentration in soils [27].

**Table 3:** The average concentrations of heavy metals in soil and rice.

Experimental Province	Soil (mg·kg^−1^)				Rice Grain (mg·kg^−1^)			
	Cd	Hg	Pb	Cr	Cd	Hg	Pb	Cr
**Hubei**	0.43 ± 0.26 ^a^	0.50 ± 0.24 ^b^	23.16 ± 6.74 ^a,b^	81.24 ± 65.75 ^a^	0.09 ± 0.10 ^a^	0.012 ± 0.01 ^a^	0.22 ± 0.06 ^a^	5.52 ± 9.65 ^a^
**Hunan**	1.12 ± 0.92 ^b^	0.15 ± 0.17 ^a^	29.00 ± 21.33 ^b^	69.00 ± 23.44 ^a^	0.41 ± 0.54 ^b^	0.014 ± 0.01 ^a^	0.33 ± 0.12 ^b^	4.91 ± 10.88 ^a^
**Jiangxi**	1.20 ± 1.24 ^b^	0.13 ± 0.09 ^a^	20.06 ± 10.02 ^a^	75.34 ± 26.79 ^a^	0.34 ± 0.24 ^b^	0.013 ± 0.02 ^a^	0.29 ± 0.11 ^b^	10.66 ± 24.02 ^a^

Note: Results are represented as mean ± standard deviation; different letters ^a, b, c^ in the same column denote that the differences are statistically significant (LSD, *p* < 0.05).

Soil properties have a considerable influence on the environmental chemistry properties and distribution of heavy metals [28,29]. The soil organic matter (SOM) concentration and soil pH were determined for all soil samples (Table 2). The soil pH values in the samples from Hubei, Hunan, and Jiangxi Provinces were in the range of 4.13–7.02, 3.90–6.95, and 3.79–6.29, respectively, with average values of 6.05, 5.92, and 5.20, respectively. The average SOM content in the soil samples from Hubei Province was 23.2 mg·kg^−1^, ranging from 7.57 to 41.96 mg·kg^−1^; these values are similar to those obtained by Wang *et al.* [30]. The average SOM content for the soil samples from Hunan Province was 32.26 mg·kg^−1^, ranging from 8.25 to 57.78 mg·kg^−1^. The SOM content in the soil samples from Jiangxi Province ranged from 7.57 to 53.65 mg·kg^−1^, with an average value of 31.57 mg·kg^−1^. The soil pH and SOM contents varied between soil samples from the different provinces.

### 3.2. Heavy Metal Content in Rice Grains

Table 2 presents the heavy metal concentrations in the rice grain samples. The concentrations of four heavy metals in the rice grain from Hubei Province exceeded the National Food Hygiene Standard values [23]. Specifically, the Cd concentrations in rice grain from sites 30 and 31 exceeded the National Food Health Standards of China [23] (Cd ≤ 0.2 mg·kg^−1^), whereas the Hg concentrations in rice grain samples from sites 20, 21, and 29 exceeded the National Food Health Standards (Hg ≤ 0.02 mg·kg^−1^). The Pb concentrations in 17 samples exceeded the National Food Health Standards (Pb ≤ 0.2 mg·kg^−1^), and the Pb and Cr standards were exceeded in some samples. The Cr concentrations in 19 samples were 1 to 12 times greater than the National Food Health Standards (Cr ≤ 1 mg·kg^−1^). The limits for Cd, Hg, Pb, and Cr were exceeded in 6.06%, 9.09%, 51.52%, and 57.58% of the samples, respectively. 

The concentrations of Cd, Hg, Pb, and Cr in rice samples from Hunan Province exceeded the thresholds of the National Food Hygiene Standard for rice grain (Table 1). The Cd threshold value was exceeded in 17 tested rice grain samples, whereas the threshold for Hg was exceeded in six samples. The Pb concentrations in all except two samples exceeded the National Food Hygiene threshold values. In the samples that exceeded the threshold value, the Pb concentrations ranged from 0.17 to 0.68 mg·kg^−1^. The Cr concentrations were below the threshold value in 10 samples; the concentrations in the remaining samples were 2–9 times greater than the standard value. The threshold values for Cd, Hg, Pb, and Cr were exceeded in 42.50%, 15%, 95%, and 75% of all rice grain samples, respectively. 

The concentrations of four heavy metals in the rice grain samples from Jiangxi Province were not below the National Food Sanitation Standards. As shown in Table 2, the National Food Sanitation Standards for Cd, Hg, Pb, and Cr were exceeded in 18, five, seven, and 18 samples from Jiangxi Province, respectively. Among the tested samples, the maximum Cd concentration was 0.96 mg·kg^−1^, which was nearly five times greater than the national limit. The maximum concentration of Cr was 93.74 mg·kg^−1^, whereas the national health standard is 1.0 mg·kg^−1^. Of the samples collected from Jiangxi Province, 42.50%, 15%, 95%, and 75% exceeded the thresholds for Cd, Hg, Pb, and Cr, respectively.

There is a clear regional pattern in the pollution: some regions are specifically polluted by certain metals. Compared with other regions, the Cr detection rate in rice grain samples was higher in Zhongxiang, Shayang and Wuhan of Hubei Province, whereas the detection rate of Pb was higher for Zhongxiang and Wuxue. Cd pollution of rice grains was more serious in Yiyang, Hengyang, and Zhuzhou of Hunan Province than in other locations. The results of this study are similar to those found by Ming *et al.* [31]. The Cd pollution of rice grain samples was more serious in some locations of Jiangxi Province than others; this trend was also confirmed by Zhang *et al.* [32].

The average concentrations of heavy metals in the rice grain samples are presented in Table 3. The average concentrations of Cd, Hg, Pb, and Cr in the rice grain from Hubei Province were 0.09, 0.012, 0.22, and 5.52 mg·kg^−1^, respectively. The average concentrations of Cd, Hg, Pb, and Cr in the rice grain from Hunan Province were 0.41, 0.014, 0.33, and 4.91 mg·kg^−1^, respectively. The average concentrations of Cd, Hg, Pb, and Cr in the rice grain from Jiangxi Province were 0.34, 0.013, 0.29, and 10.66 mg·kg^−1^, respectively. Comparing the samples from the three provinces (Table 2 and Table 3), The Cd and Pb accumulation of the rice grains among the test samples was in the order of Hunan = Jiangxi > Hubei. The Cr contamination of the soils among the test samples from the three provinces showed no significant difference. The accumulation of heavy metals in rice grains is likely influenced by more than the heavy metal accumulation in soil. Studies have shown that heavy metal contamination in rice plants may also be due to air pollution, sewage water irrigation, and pesticide spraying [33,34,35].

### 3.3. Relationship between Heavy Metal Concentrations in Soil and Rice Grains

The concentrations of Cd, Hg, Pb, and Cr in soils and rice grain from the Yangtze River region are shown in Table 4. The results show that 70.90%, 29.7%, 0%, and 0% of the 101 soil samples collected in the study area were polluted by Cd, Hg, Pb, and Cr, respectively. There was considerable accumulation of Cd in the soil. The concentrations of Cd, Hg, Pb, and Cr in rice grain from the Yangtze River region ranged from 0.01 to 1.81, 0.00 to 0.09, 0.12 to 0.68, and 0.23 to 93.74 mg·kg^−1^, respectively. The average Cd, Hg, Pb, and Cr concentrations in rice grain were 0.29, 0.01, 0.25, and 6.70 mg·kg^−1^, respectively. Of the samples that had excessive heavy metal concentrations, the number of samples in which the Pb concentrations exceeded the standards was greatest, followed by Cr and Cd; these results indicate that rice grain was seriously polluted by Cd and Cr. Compared with the trace metal contents in agricultural soils in other areas in China [36] and in other countries [37], the mean concentrations of Cd and Cr in the Yangtze River region were generally higher, whereas Pb was generally lower in our study.

**Table 4:** The concentrations of four heavy metals (Cd, Hg, Pb, Cr) in soil and rice grain from the Yangtze River of China.

Items	Soil				Rice Grain			
	Cd	Hg	Pb	Cr	Cd	Hg	Pb	Cr
Number	101	101	101	101	101	101	101	101
Max (mg·kg^−1^)	4.64	1.46	127.56	231.02	1.81	0.09	0.68	93.74
Min (mg·kg^−1^)	0.10	0.01	7.64	13.52	0.01	0	0.12	0.23
Overall mean (mg·kg^−1^)	0.79	0.18	24.62	74.76	0.29	0.01	0.25	6.70
Standard deviation (mg·kg^−1^)	0.94	0.25	15.27	44.13	0.39	0.01	0.11	15.42
Median (mg·kg^−1^)	0.55	0.16	21.55	64.62	0.10	0.01	0.25	2.66
Percentage of excessive amount	70.90%	29.7%	0%	0%	37%	16%	60%	70%

Our results showed that the soil was not polluted by Pb and Cr, but the concentrations of Pb and Cr exceeded the threshold values in rice grain; this may be related to sewage water irrigation on the soil surface, heavy metal deposition from the air, fertilizers and the variety of rice being cultivated [25,38,39,40]. Many studies have shown that transport emissions can influence the concentrations of heavy metals in plants [41,42,43,44], particularly Pb concentrations [45]. Furthermore, the rice grain grown in Cd-contaminated soil was not polluted by Cd; this may be related to the varieties of rice being cultivated [40].

**Figure 2:**
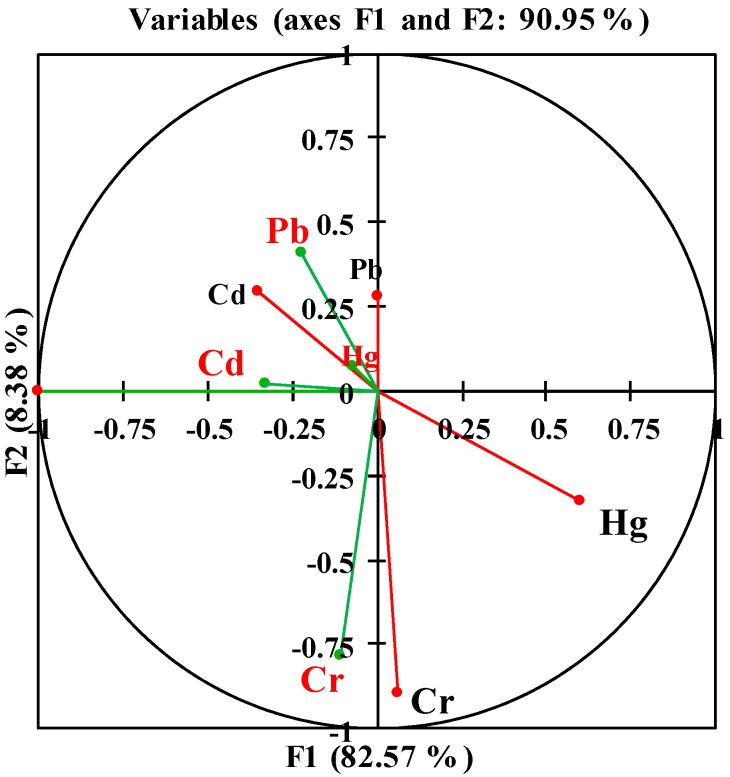
Ordination diagram based on the CCA of the heavy metal (Cd, Hg, Pb, and Cr) in soil (in black) and heavy metal (Cd, Hg, Pb, and Cr) in the rice grain (in red).

CCorA was applied to study the relationship between the heavy metal concentrations in rice grain and the heavy metal concentrations in soil. The CCorA results are plotted in Figure 2. The first two factors (F1 and F2) in the multivariate analysis represented 90.95% of the total variance and showed that the Cd and Pb concentrations in rice grain are positively related with the Cd and Pb concentrations in the corresponding soil, as expected. However, the results also showed that the Hg concentrations in soil are positively related to the Cr concentrations in the soil and that the concentrations of Cd and Pb in soil are positively correlated. The concentration of Hg in soils was found to be influenced by the accumulation of Pb and Cd in the rice grain. These results illustrate that the interactions among the heavy metal elements may also directly influence the migration and accumulation of heavy metals in soil or paddy rice. This conclusion was also obtained by Adams *et al.* [46].

The heavy metal concentrations in rice grain and soil do not exhibit a one-to-one correlation. Rogival *et al.* [47] showed that the concentrations of heavy metals in soil were not the sole determinant of heavy metal accumulation in crops. Thus, the concentrations of heavy metals in soils were not the sole determinant of the heavy metal accumulation in the corresponding rice grain, which suggests that other factors may influence heavy metal accumulation in rice grain.

### 3.4. Relationship between Soil Properties and Heavy Metal Concentrations in the Soil-Rice System

**Figure 3:**
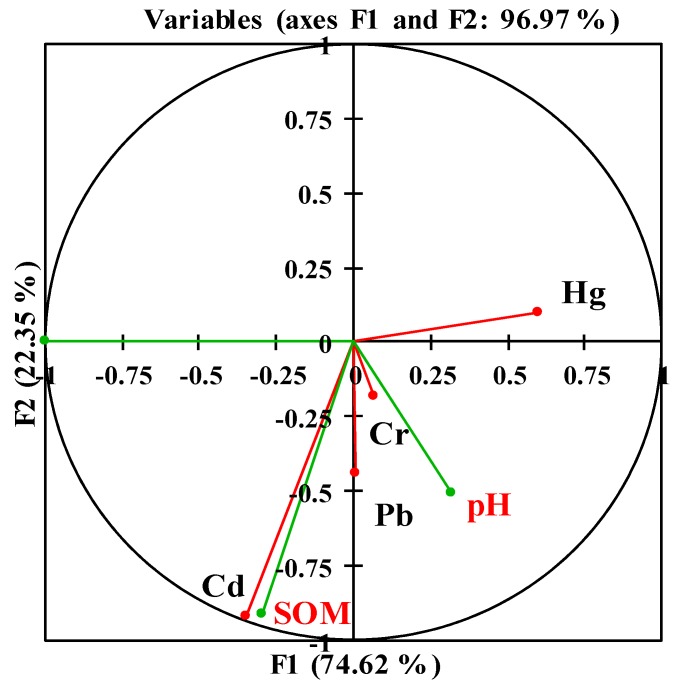
Ordination diagram based on the CCA of soil properties (in black) and heavy metals (Cd, Hg, Pb, and Cr) in the soil (in red).

**Figure 4:**
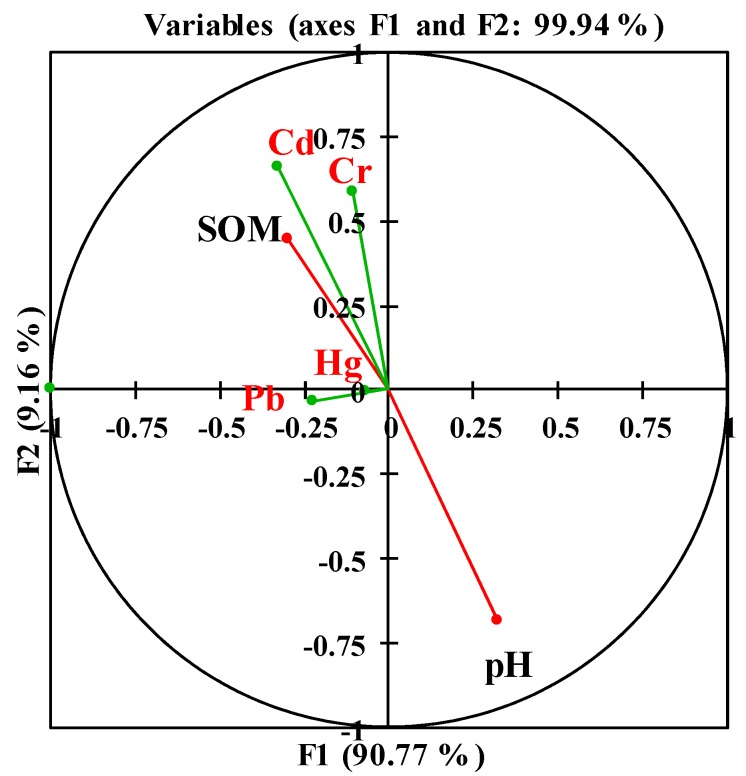
Ordination diagram based on the CCA of soil properties (in black) and heavy metals (Cd, Hg, Pb, and Cr) in rice grain (in red).

The pH value and SOM content are important physicochemical properties of soil. Numerous studies have shown that soil pH and SOM control the accumulation of heavy metals in soil and plants [48,49]. CCorA was used to study the relationships between the heavy metal concentrations in soil and the soil properties (soil pH and SOM), as well as the relationships between the heavy metal concentrations in rice grain and soil properties. The results are shown in Figure 3 and Figure 4. As shown in Figure 3, the first two factors (F1 and F2) in the multivariate analysis represented 96.97% of the total variance. The relationships between the soil properties and heavy metal concentrations in soil are not straightforward. There was a positive relationship between the soil pH values and Cr concentrations in soil. The SOM content was positively correlated with the Cd concentrations in soil samples. However, the relationships between Pb/Hg in soil and SOM content were not significant.

As shown in Figure 4, the first two factors (F1 and F2) in the multivariate analysis represented 99.94% of the total variance. The concentrations of Cd, Hg, Pb, and Cr in rice grain were negatively correlated with the soil pH value. The concentrations of Cd, Cr and Hg in rice grain were positively correlated with SOM content. The Cd and Cr concentrations in rice grain tended to be positively correlated with SOM content, which may favor the retention of these metals in the soil.

Table 2 and Table 3 illustrate that rice grain samples that had high Cd concentrations generally had soil pH values less than 5.0. In general, as the pH value decreased, the absorption of Cd in rice grain increased. When the soil pH was higher than 6.0, the corresponding Cd concentration in the rice grain was within the national limits for rice grain, even when the Cd concentration in the soil was relatively high.

Soil pH has the ability to significantly alter the chemical speciation of the heavy metals in soil and influences the availability of heavy metals in soil and the ability of plants to absorb heavy metals [50,51,52]. Our study demonstrated that soil pH has an influence on heavy metal (Cd, Cr and Hg) concentrations in rice grain; as the soil pH value increased, the heavy metal concentrations in the rice grain decreased to a certain extent. This finding is similar to the findings of other studies [49,51,53] and illustrates that low pH may result in increased solubility and a high availability of heavy metals in soil [53], influencing the absorption of heavy metals by rice grain. Our results showed that SOM could significantly influence the Cd concentrations in soil, thereby affecting the absorption of Cd in rice grain. The effect of SOM on Cd could be due to the higher solubility of Cd in soils with increases in SOM. This result was in contrast to a previous study by Ghosh [54].

The migration and transformation processes of heavy metals in soil-crop systems are extremely complex, and many factors are involved. The total amount of heavy metals in soil, soil pH and SOM are major controlling factors in the migration of heavy metals from soil to plants. Numerous studies have indicated that differences in soil type, interactions between the elements, soil electrical conductivity, clay content, nutrients, enzyme activity, and other physical and chemical properties could directly influence the migration and accumulation of heavy metals in soil, as well as the absorption and accumulation of heavy metals in paddy rice [46,49,54,55,56]. Other possible influences include the type of paddy field examined, irrigation water, the atmosphere, and soil microbiological activity [57,58].

## 4. Conclusions

Based on the guideline values for heavy metal pollution, some paddy fields of the Yangtze River of China showed Cd and Hg contamination, and rice grains were polluted by Cd, Hg, Pb, and Cr. Canonical correlation analyses showed that the elements Cd and Pb in rice are related to the elemental concentrations in the corresponding soil, and the interactions among the elements Cd, Pb and Cr in soil directly influences the accumulation of heavy metals (Hg, Cd, Pb and Cr) in paddy soil and rice. Furthermore, soil pH and SOM were the important factors controlling the accumulation of Cd, Pb and Cr in soil and rice. These findings have important implications for the development of pollution prevention and reduction strategies to reduce heavy metal pollution for regions. This study can serve as a reference for the regional management and environment quality assessment of the study area. In further work, some experiment, which combined with soil properties, could be used for rational site-specific management in paddy fields.
